# The relationship between vasculogenic mimicry and epithelial‐mesenchymal transitions

**DOI:** 10.1111/jcmm.12851

**Published:** 2016-03-29

**Authors:** Qiqi Liu, Lili Qiao, Ning Liang, Jian Xie, Jingxin Zhang, Guodong Deng, Hui Luo, Jiandong Zhang

**Affiliations:** ^1^ Department of OncologyShandong University School of MedicineJinanShandong ProChina; ^2^ Department of Radiation OncologyQianfoshan Hospital Affiliated to Shandong UniversityJinanShandong ProChina; ^3^ Department of OncologyWeifang Medical CollegeWeifangShandong ProChina

**Keywords:** vasculogenic mimicry, epithelial‐mesenchymal transition, cancer, antitumour, tumorigenesis

## Abstract

Vasculogenic mimicry (VM) is a vascular‐like structure which can mimic the embryonic vascular network pattern to nourish the tumour tissue. As a unique perfusion way, VM is correlated with tumour progression, invasion, metastasis and lower 5‐year survival rate. Notably, epithelial‐mesenchymal transition (EMT) regulators and EMT‐related transcription factors are highly up‐regulated in VM‐forming tumour cells, which demonstrated that EMT may play a crucial role in VM formation. Therefore, the up‐regulation of EMT‐associated adhesion molecules and other factors can also make a contribution in VM‐forming process. Depending on these discoveries, VM and EMT can be utilized as therapeutic target strategies for anticancer therapy. The purpose of this article is to explore the advance research in the relationship of EMT and VM and their corresponding mechanisms in tumorigenesis effect.

## Introduction

It is wildly acknowledged that tumour development and growth need sufficient blood supply. Former researches considered that tumour angiogenesis as the unique way to satisfy the blood acquirement. But recent findings indicate that there exists a novel pathway called vasculogenic mimicry (VM), a vascular‐like structure which can mimic the embryonic vascular network pattern to nourish the tumour tissue. When utilizing periodic acid‐schiff stain to identify VM, periodic acid‐schiff‐positive labelling verified that channels are inner‐lined by tumour cells instead of endothelial cells 1. Currently, the certain mechanism of VM formation is still under discovery, the main mechanism is correlated with the following aspects: ECM remodelling, certain tumour microenvironments and cancer stem cells (CSCs) or dedifferentiated stem‐like cells. As VM‐forming cancer cells are able to alter their cell markers and form vessel‐like structures similar with embryonic vasculogenesis pattern, VM is commonly considered as an example of aggressive tumour cells with remarkable differentiation plastic capacity, and VM‐positive aggressive tumour cells are induced to increase expression of genes associated with undifferentiated embryonic phenotypes. Considering the phenomenon that tumour cells are able to transdifferentiate into endothelium‐like cells which are mesenchymal cells, it can be demonstrated that epithelial‐mesenchymal transition (EMT) is vital in VM formation and tumour progression. However, the up‐regulation of EMT‐associated transcription factors can also contribute in VM‐forming process 2, 3, 4. The purpose of this article is to explore the most recent research in the relationship of EMT and VM and their corresponding mechanisms in tumorigenesis effect. This thesis consists of three parts, the first part introduces the conceptions, the second part studies the corresponding mechanisms, and the last part is perspectation and conclusion.

## Background of VM

Vasculogenic mimicry is firstly described by Maniotis when he found that in a tubular structure, tumour cells are pasted on the basement membrane in aggressive melanoma in 1999 5. Frenkel *et al*. in 2008 demonstrated blood circulation in VM tube in a choroidal melanoma patient using lasers canning confocal angiography 6. This confirmed VM as a novel pattern to provide nutrition for malignant tumour without conventional vasculogenesis or angiogenesis. Notably, there actually exist three‐stage blood supply patterns in tumour: VM, mosaic vessels and endothelium‐dependent vessels 7, 8. All these three patterns can provide blood supply for tumours. The model considers VM as the dominant blood supply pattern at the early stage during tumour growth. As a result, endothelial cells would proliferate and differentiate to supply nutrition for tumour mass expansion, and then the mosaic vessels act as a transitional pattern. At the advanced stage of malignant tumour, endothelium‐dependent vessels would be the significant way to provide the blood supply instead of VM and mosaic vessels. Thus, VM served as the major role to nourish the tumour mainly at the early stage of tumour growth. Vasculogenic mimicry forms an extracellular matrix (ECM)‐rich network and exposes tumour cells directly to blood flow, allows them to enter the microcirculation environment and metastasis to other organs, thus plays a crucial role in tumour growth and metastasis 4. Vasculogenic mimicry has been observed in several of malignant tumours, such as breast cancer, prostate cancer, liver cancer, malignant glioma, melanoma and so on 9. Compared with malignant tumour patients without VM, patients with VM have a worse prognosis 10, which may because of the direct connection of tumour cells and blood flow. In addition, VM is also one of the obstacles in the poor treatment of anti‐angiogenic drug for malignant tumour endothelial cells. Anti‐angiogenesis drugs can inhibit new vessels formation by human vascular endothelial cells *in vitro*, while under the same conditions fails to affect VM formation.

## Background of EMT

Epithelial‐mesenchymal transition is a dynamic biological process that converts epithelial cancer cells into dedifferentiated cells with additional mesenchymal properties. At the beginning, EMT is recognized as a crucial embryogenesis process in embryonic development and several of pathological processes including wound healing and organ fibrosis 11. Epithelial‐mesenchymal transition is characterized by loss of contact inhibition, remodelling of ECM, and reorganization of cytoskeleton 12, 13. The hallmarks of EMT include loss of epithelial traits and the gain of mesenchymal phenotypes 14. Epithelial‐mesenchymal transition is also accompanied by increased expression of transcription factors including Twist, Snail, zinc‐finger E‐box binding homeobox 1 (ZEB1) and ZEB2, which can bind to E‐cadherin promoter and suppress its transcription activity 15. Upon initiation of EMT, loss of E‐cadherin is capable to enhance invasion capacity of carcinoma cancer cells during progression. This finding verified the crucial role of EMT in the conversion from benign tumour to highly aggressive cancer *via* providing highly malignant cells which can escape from immune system, apoptosis process, and conventional targeted therapies. Epithelial‐mesenchymal transition can be modulated by several of transcription factors including Twist, Snail, Slug, Sox4, Zeb4 and FoxC2 that repress E‐cadherin expression and initiate the characteristic morphological changes 16, 17.

Transforming growth factor (TGF‐β) is one of the most important EMT inducers 18. The mechanism is *via* both Smad‐dependent and ‐independent pathways including p38 MAPK. P38 MAPK can also be activate by Ras and Wnt which act with TGF‐β synergistically, while the Smad pathway is unique to TGF‐β signalling 19. Hepatocyte growth factor plays a crucial role in initiating EMT *via* both PI3K/Akt pathway and RAF‐MEK‐ERK pathway 20, 21. The PI3K/Akt pathway can modulate the expression of MMPs to inhibit cancer metastasis 22. Moreover, activation PI3K/Akt pathway has been considered as a central feature of EMT *via* modulating key EMT factors such as Snail 23. Besides, VEGF is capable to initiate the EMT process to regulate the invasion and metastasis of malignant cancers 24, 25. Epithelial‐mesenchymal transition plays crucial roles in VM formation. Epithelial‐mesenchymal transition‐like phenotype can be induced by hypoxic microenvironment which is of great significance in inducing VM in cancer cells. This phenotype is characterized with increased motility and invasiveness, and fibroblast‐like cell‐cell adhesion, which allows the cells to break free from the primary tumour site and metastasize at distant sites 26, 27. Moreover, it is demonstrated that EMT‐related transcription factors are up‐regulated in VM‐forming tumour cells. Zinc‐finger E‐box binding homeobox 1 expression is increased in VM‐positive malignant tumours, and its knockdown would lead to a decrease in VM and restoration of certain epithelial phenotypes 28. The decrease of E‐cadherin and increase of vimentin can also been seen in the ZEB1‐positive group. The inhibition of Twist1 expression by the short hairpin RNA can significantly suppress the formation of VM in hepatocellular carcinoma (HCC) 4. The Bcl‐2/Twist1 complex facilitating the nuclear transport of Twist1 leads to transcriptional activation of a wide range of genes, which may increase tumour cell plasticity, metastasis, and VM formation in HCC 29. These findings indicated that EMT is involved in VM formation and may provide a therapeutic target for anti‐angiogenesis therapy 30.

## VM, EMT and CSCs

Recent discoveries found that VM formation is correlated with CSC. Cancer stem cell is a small portion of cells which are able to differentiate/transdifferentiate into several of cell types to resemble normal stem cells in function and phenotype 30, 31. Cancer stem cells exist in many malignant tumours including breast, colon, prostate cancer, melanoma and glioblastoma. The reversible self‐renewal and multipotent differentiative properties are the main characteristics of CSCs. Many researchers have found the linkage between tumour cells and the CSC phenotype undergoing EMT. The expression of surface stem‐like marker CD44, as well as genes involved in the maintenance of a stem cell phenotype is also much higher in these cells. The EMT phenotype of this cell population is characterized by the gain of N‐cadherin and loss of E‐cadherin expression. Twist1 can also support the fact that EMT is correlated with cellular stemness. Twist1 is a significant EMT regulator that can directly modulate the stemness factor Bmil, which is inevitable in the self‐renewal capacity of normal stem cells 32. In tumour cells lines, Twist1 and Bmil overexpression would result in the EMT characteristic acquisition and stem cell markers induction, thus to enhance the capacity of tumour invasion and metastasis. Reports also illustrated that invasive malignant tumour cells with CSC‐like characteristics are more tumorigenic than their counterparts 33. In solid tumour, CSCs posses the differentiation plasticity and normal stem cells’ properties to be involved in tumour growth and vascularization. For example, VM channel is formed by the transdifferentiation of malignant stem cells subset in aggressive malignant melanoma. Cancer stem cells in endothelial differentiating medium are able to differentiate into endothelial cells, which are capable to form both vessels and tumour 34. The multipotent differentiation phenomenon of CSCs and their involvement in VM formation can also be found in human B‐cell lymphomas, multiple myeloma, neuroblastoma and glioblastoma 35. Thus, these discoveries demonstrated that CSCs participate in VM formation of tumours. In this progress, CSCs would form VM aggregation in tumorigenic microenvironment firstly, and then CSCs would differentiate or transdifferentiate, as well as line‐up to form branching tubes and lumens which provides nutrition for tumour mass, resembling vascular network. Finally, the tubes extend and merge with vessels from angiogenesis or vascularization, and conduct blood cells. In addition, EMT is widely acknowledged to be involved in cancer invasion. Besides its contribution to invasion, EMT can also promote forming the secondary metastasis location of disseminated cancer cells as a result of its self‐renewal capability 4, 33. Further evidence shows that EMT regulators, including Twist and Snail, and EMT‐related transcription factors are highly up‐regulated in VM‐forming tumour cells 5. For example, Twist1 up‐regulation would significantly enhance the invasiveness and metastasis capacity of tumour cells, as well as promote VM formation and stimulate the secretion of VM‐associated molecules including VE‐cadherin. All these factors verified the relationship between EMT, CSCs and VM‐forming cells. Na *et al*. 5 showed that VM‐forming cells are characterized by the expression of various factors responsible for mediating EMT at the molecular level in tumours, which can also be a proof that EMT process correlated with CSCs is involved in VM formation.

## Molecules correlated with EMT and VM

It is acknowledged that EMT makes contribution in VM formation, so adhesion molecules and other factors which can modulate EMT process can regulate VM formation. This mechanism provides a therapeutic target for anti‐angiogenesis therapy. The correlation between EMT transcription factors and VM is summarized in Table 1.

**Table 1:** The correlation between EMT transcription factors and VM

**EMT transcription factors**	**Functions in VM formation**	**References**
Twist	Twist represses E‐cadherin and upregulates N‐cadherin and vimentin. Downstream targets of Twist include platelet‐derived growth factor receptor‐a, Akt2, Snail1, and Snail2.	4, 41–54
ZEB1	ZEB1 downregulates E‐cadherin, decreases vascular endothelial cadherin and Flk‐1 expression, as well as induces EMT and stemness maintenance in VM‐positive cancer cells.	57, 60–63
Snail	Snail1 interacts with Ezh2 and Suz12, recruit polycomb complex 2 to repress CDH1 which can encode E‐cadherin.	64–69
Slug/Snal2	The role of slug in promoting VM formation is related to an increase in CSCs subpopulation generated from EMT.	70–74

EMT, epithelial‐mesenchymal transition; VM, vasculogenic mimicry; ZEB1, Zinc Finger E‐Box Binding Homeobox 1.

### Twist1/Bmi1

Twist, a transcription factor, is a major EMT inducer which plays a predominate role in cancer metastasis process *via* several of signal pathways 4. Twist is a basic helix‐loop‐helix (bHLH) transcription factor, encoded by the Twist1 gene located on human chromosome 7p21. Twist is also known as Twist1, as it shares similarity with another bHLH protein named Twist‐related protein (Twist2) which has different roles 36. Downstream targets of Twist include Akt2, Snail1, Snail2 and platelet‐derived growth factor receptor‐α. The stability of Twist is of great importance in cells 37. Twist is capable to open nuclear membrane pores with the help of an accessory protein and enters the nucleus to regulate transcription of downstream genes that are involved in the process of VM 38. However, Twist2 share several of similar functions including their regulation of haematological malignancies and their role in cancer progression and metastasis with Twist1 39, 40, 41. Studies found that Twist1 and Twist2 were both up‐regulated in metastasis‐associated colon cancer. Silencing of either Twist1 or Twist2 inhibited VM, suggesting that activation of either molecule is sufficient in inducing VM formation. Twist induced EMT and metastasis are associated with poor survival in several of cancers 42, 43, 44. For example, Twist is correlated with nodal metastasis in breast carcinoma, which is related with more high‐grade malignant tumours. Twist suppresses oestrogen receptor expression by recruiting DNA methyltransferase of the oestrogen receptor promoter and interacting with histone deacetyltransferase 1 45. Twist1 can also counteract the posttranslational modifications of p53 *via* binding to the Twist box on the C‐terminus of p53, as well as facilitate its degradation to inhibit the cell cycle arrest and apoptotic progress induced by p53 46. In human breast and cervical cancer, Twist overexpression is able to transform cancer cells into CSC phenotypes coupled with high CD44 expression, increased aldehyde dehydrogenase 1 activity, and no or little CD24 expression independent of EMT formation mechanisms 47, 48, 49. Activation of Akt/β‐catenin signalling pathways, as well as expression of hypoxia inducible factor 1‐α (HIF1‐α) and NF‐κB are of great significance for Twist to maintain the EMT‐associated CSCs. However, Twist suppressors like Prospero‐related homeobox 1 (Prox1), a member of the homeobox transcription factor family, is able to inhibit proliferation, migration and invasive capacity of cancer cell lines, as well as inhibit EMT progress, thus providing novel useful strategies for cancer treatment and prevention. Knockdown of Twist1 by siRNA also shows remarkable effect on reduction of apoptosis and cell death of cancer cell lines.

Notably, study shows that hypoxia is capable to induce Twist expression and VM formation in malignant cancers 50. Hypoxia up‐regulates the expression of HIF‐1, which can further combine with the promoter of Twist1 to initiate its transcription, and thus induce the occurrence of EMT. Hypoxia inducible factor 1α is able to initiate EMT *via* activating Snail thus repressing E‐cadherin expression, as well as transactivating MMP9 in carcinomas 51. As co‐expression of HIF‐1α, ZEB1 and decreased expression of E‐cadherin is considered as a significant marker to predict the invasion and migration capacity of malignant tumour cells. These findings would provide a molecular basis for promotion of the invasive cancer phenotype by HIF‐1α overexpression. Bmi1 is one of the polycomb group members and can be directly regulated by Twist1. Both of them are significant in promoting cell stemness, and their co‐overexpression would promote cancer cells’ tumour‐initiating capability and VM formation. Accompanied by the up‐regulation of Twist1 and Bmi1, E‐cadherin expression in the two cells lines is down‐regulated and vimentin, an indication of EMT up‐regulated in hypoxia condition. Vasculogenic mimicry‐associated makers including VE‐cadherin, as well as CSC markers including CD44 and Oct4 are also up‐regulated following Twist1‐Bmi1 cooperation. These facts means that cell stemness can also be induced under hypoxia condition. The epithelial cells could firstly obtain high levels of stemness, and in subsequent differentiate into mesenchymal‐like cells. Ectopic expression of Bmi1 would cause EMT which can subsequently induce stem‐like cells 52. The mutual promotion of these two processes is the basis of VM formation. This verified that the effect of hypoxia on VM formation is *via* Twist1‐Bmi1 connection, which induced EMT and stemness. So, hypoxia is able to promote Twist up‐regulation and in addition, targeting Twist or Twist‐related molecules provides a novel therapy for tumour prevention and treatment by abolishing the CSC phenotypes, reducing cancer resistance and recurrence, as well as sensitizing them to drugs, thereby improving patient survival.

Bcl‐2 and its family members participate in anti‐apoptosis process, protein modification and complicated cell metabolism processes. Bcl‐2 exhibits an increased expression similar with Twist1 in hypoxia condition 29. This phenomenon indicated that Bcl‐2 and Twist1 possibly acted during the stress phase in the same cell and followed similar kinetics. In tumour tissues, Bcl‐2 expression in the nucleus is correlated with poor prognosis. Bcl‐2 could form a complex with Twist1 to synergistically promote the transcription of downstream target genes, which can lead a cascade changes in proliferation, migration, metastasis and vasculogenesis in malignant tumours. The effect of the synergetic action has a better outcome compared to Twist1 alone. In addition, Bcl‐2 is capable to up‐regulate VE‐cadherin expression and promote the formation of VM pattern in the three‐dimensional matrigel 53. Bcl‐2 has the ability to activate the promoter for VE‐cadherin transcription regulation. This provides a novel mechanism for cancer development mediated by Twist1, and provides a foundation for the design of a novel inhibitor for EMT and angiogenesis.

### Zinc‐finger E‐box binding homeobox 1

Zinc‐finger E‐box binding homeobox 1, zinc‐finger E‐box binding homeobox 1, is one of the major EMT inducers and pro‐metastatic transcription factors 54. Zinc‐finger E‐box binding homeobox 1 is able to increase metastasis capacity and promote EMT in several of human cancers including cancer of prostate, colon, breast and pancreas 55, 56, 57. The ectopic expression of ZEB1 down‐regulates E‐cadherin and induces EMT in malignant tumour *via* binding with its conserved E‐boxes on promoter. Zinc‐finger E‐box binding homeobox 1 decreases the expression of vascular endothelial cadherin and Flk‐1, which are characteristics of endothelial cells. *In vitro*, knockdown of ZEB1 could result in epithelial phenotypes restoration and significantly inhibit migration and invitation capacity. However, stromal expression of ZEB1 increased in low‐grade endometrial carcinomas, epithelial expression of ZEB1 was found in high‐grade endometrial carcinomas, suggesting that aberrant expression of ZEB1 induces EMT, contributing to aggressive behaviours of malignant cancers. As a crucial EMT inducer, ZEB1 not only promotes malignant progression, but is also necessary for the tumour‐initiating capacity of epithelial cancer cells 55. In VM‐positive malignant tumour cells, ZEB1 expression is higher compared with the VM‐negative tumour cells and the ZEB1 expression occurred concomitantly with features of EMT. Zinc‐finger E‐box binding homeobox 1 is also able to control other important cellular functions and states including stemness and differentiation. In VM‐positive cancer cells, ZEB1 could indirectly induce stemness maintenance more efficiently. These results all suggest that ZEB1 can promote VM formation by inducing EMT in malignant tumour cells.

### Snail

Snail is of enormous significance in physiological EMT. Snail overexpression in tumour cells is able to promote VM formation induced by EMT and increased CSCs, exhibit a better capability of growth and invasion 58, 59, 60. It can be verified that snail is overexpressed in highly aggressive tumour cells but not in normal tissues. The underlying mechanisms include that Snail1 can interact with Ezh2 and Suz12, in the meanwhile recruit polycomb complex 2 to repress CDH1 which can encode E‐cadherin. The protein structure of snail has two regions including C‐terminal DNA‐binding region and N‐terminal regulatory region. Snail1 binds with its C‐terminal region E2‐box [C/A(CAGGTG)] on the promoter, or interact with histone deacetylases with its SNAG sequence in the N‐terminal region to down‐regulate CDH1 expression 61. Notably, the Snail1 mRNA translation can be activated by Y box binding protein 1 in various malignant carcinomas and there exist several of post‐translational modifications including the p21‐activated kinase 1 which can regulate the level of subcellular localization *via* Snail phosphorylation, as well as glycogen synthesis kinase 3b‐mediated phosphorylation which would facilitate the ubiquitine‐dependent Snail degradation 62, 63. All these cooperative corepressors are required for Snail to form a repressive complex inhibiting EMT and VM formation.

### Slug/Snal2

Slug (Snal2), a zinc‐finger transcription factor, was considered to be a significant mediator of Twist1‐induced EMT 64, 65. In recent researches, slug overexpression was associated with the stemness behaviour of CSCs 66. Slug can not only regulate the immunophenotype of CSCs but also mediate radioresistance and chemoresistance by inducing cancer stem‐like properties 67. Slug overexpression is correlated with the up‐regulation of vimentin expression and the down‐regulation of E‐cadherin expression, which indicates that slug is sufficient in promoting EMT. The induction of EMT can generate a population with stem cell characteristics from well‐differentiated epithelial cells and cancer cells 42, 43, 44. Notably, EMT and CSCs phenotype induced by slug overexpression could be linked to each other. Slug overexpression is correlated with poor prognosis through promoting VM which can be induced by EMT and the generation of CSCs displaying the plasticity of epithelial cells. Then, the increased CSCs can transdifferentiate into different phenotype, they express angiogenic and vasculogenic markers such as VEGF and VE‐cadherin and they are able to organize pseudovascular network. Cancer stem cells in HepG2‐slug *in vivo* can further differentiate into endothelial cell‐like tumour cells to participate in the construction of tumour microcirculation. In addition, cancer stem–like cells might also directly contribute to the tumour angiogenesis by converting to endothelial cell 68. It is demonstrated that tumours in HepG2‐slug xenograft presented more vascular vessels of human tumour cell origin than HepG2 xenograft. Therefore, slug is capable to promote VM in HCC by the induction of EMT, pluripotency and CSCs‐like phenotype *in vitro*,* in vivo* and in HCC patients. Vasculogenic mimicry represents an important survival mechanism contributing to the failure of currently available angiogenesis inhibitors to fully effect tumour eradication. Thus, slug can be considered as a novel target for new therapeutic perspectives in HCC.

### MicroRNAs

MicroRNAs are gene expression regulators which are crucial in several of biological processes including cell differentiation, apoptosis, cell cycle and EMT. MicroRNAs are capable to target cancer‐related genes, result in their translational repression or degradation and consequently, act as oncogenes or tumour suppressors 69. Several microRNAs have been identified to regulate EMT. The miR‐200 family, composed of miR‐200a, miR‐200b, miR‐200c, miR‐141 and miR‐429, plays a significant role in EMT suppression mainly *via* targeting ZEB 70, 71, 72. The TGF‐β/ZEB/miR‐200 signalling regulatory network is significant in EMT regulation. MiR‐200 overexpression would up‐regulate E‐cadherin and inhibits EMT *via* targeting the transcription factors ZEB1 and ZEB2 73. Transforming growth factor‐β2 is a predominant target of the miR‐200 family and the relief of miR‐200‐mediated inhibition of TGF‐β2 increases the autocrine effect of TGF‐β, which is significant in EMT progression. The miR‐200 expression can also change the tumour microenvironment to inhibit EMT and metastasis in malignant carcinomas 74. There are also many other microRNAs correlated with EMT regulation. MiR‐205 can act with miR‐200 synergistically to suppress ZEB and suppress EMT 75. MiR‐205 can also sustain the differentiation of epithelial cells in mammary gland. MiR‐148a, miR‐505 and miR‐1207‐5p, induced by growth factors, act as a negative feedback loop of EMT 69, 76. The three microRNAs can be served as EMT and metastasis inhibitors by repressing expression of EMT‐related molecules including Snail, fibronectin and β‐catenin. Snail/miR‐34 is another double‐feedback loop in which increased snail expression can suppress miR‐34 in TGF‐β‐induced EMT. These double‐feedback loops are proposed to controls the epithelial plasticity and stimulate EMT in tumour progression 77, 78. MiR‐29b suppresses EMT and metastasis progress in prostate cancer 76, 79. MiR‐148a can negatively regulate Met/Snail signalling and prevent EMT and metastasis in hepatoma cells 76, 80. Therefore, it is verified that microRNAs function as gene expression buffers and take part in maintaining robustness in EMT process through feedback regulation. The major functions of miRNAs in VM are illustrated in Table 2.

**Table 2:** The major functions of miRNAs in VM

miRNAs	Role of EMT‐regulating miRNAs	References
The miR‐200 family	Target ZEB1/2 *via* the TGF‐β/ZEB/miR‐200 pathway, as well as change the tumour microenvironment to inhibit EMT and metastasis.	69, 70, 71, 72, 73, 74
MiR‐205	Act with miR‐200 synergistically to suppress ZEB and suppress EMT, as well as sustain the differentiation of epithelial cells in mammary gland.	74, 75
MiR‐148a, miR‐505 and miR‐1207‐5p	Act as EMT and metastasis inhibitors by repressing expression of EMT‐related molecules including Snail, fibronectin and β‐catenin.	69, 76
Snail/miR‐34 feedback loop	Control the epithelial plasticity and stimulate EMT in tumour progression.	77, 78
MiR‐29b	Suppress EMT and metastasis progress in prostate cancer.	76, 79
MiR‐148a	Negatively regulate Met/Snail signalling and prevent EMT and metastasis in hepatoma cells.	69, 76, 80

EMT: epithelial‐mesenchymal transition; ZEB: zinc‐finger E‐box binding homeobox; TGF‐β: transforming growth factor‐β.

## Perspectives on cancer treatment

Epithelial‐mesenchymal transition‐related factors are transcription factors which are crucial in pathologic cancerous progression and difficult for cancer therapeutics to target. MicroRNA is capable to inhibit positive regulators of the EMT program with stability and specificity. Augmentations of negative transcription factors can also be an alternative to revert EMT, such as DEAR1 or KLF17 in breast carcinoma. More researches are needed for antibody or small‐molecule tyrosine kinase inhibitors to target TGF‐β, Notch, and the snail or Wnt/β‐catenin pathways. Doxycycline has been used in combination with targeted drugs in clinical trials with patients with advanced cancer 81. Doxycycline is a semi‐synthetic tetracycline which can inhibit MMP activation and cell proliferation, as well as interfere with tumour‐related protein synthesis in mammalian cells. Doxycycline has a strong inhibitory effect on malignant cells especially NCI‐H446 and A549 cells. Doxycycline is capable to up‐regulate the level of E‐cadherin levels and down‐regulate the expression of vimentin protein. It has been revealed that doxycycline inhibits EMT‐related transcription factor activity and that doxycycline exerts its antitumour effect by interfering with tumour cell EMT. These findings show that doxycycline acts upstream of EMT‐related signal transduction to inhibit a wide range of cellular functions 81, 82, 83. In addition, methacycline appear to have similar inhibitory effects on TGF‐β1‐induced EMT in both A549 cells and lung epithelial cells compared with doxycycline, although doxycycline is not as potent as methacycline 84. Methacycline affects Smad signalling indirectly through regulation of non‐Smad signalling, likely through TGF‐β1 itself and TGF‐β receptor levels that then further attenuates EMT and fibrosis *in vivo*. Thus, doxycycline and methacycline can be considered to be alternative therapies for persistent carcinoma. Cancer stem cells are implicated in VM formation and served as a promising target for anticancer therapies. The existence of CSCs is also responsible for the low survival rate of patients with aggressive tumours. After chemotherapy and radiotherapy, only a small proportion of CSCs are capable to induce recurrence. Furthermore, EMT is also involved in the acquisition of CSC properties, the combination of targeting EMT and CSCs may be beneficial for anti‐VM formation therapy, decreasing invasion and metastasis, and improving the survival rate of patients. As is discussed earlier, VM served as a major role to nourish tumour masses mainly at the early stage of tumour growth and CSCs are able to differentiate/transdifferentiate into branching lumens. As there are no normal endothelial cells in VM, little effect can be exhibited on VM with traditional anti‐angiogenesis drugs such as endostatin and angiostatin 85. Moreover, decreased blood vessel density caused by anti‐angiogenic therapy would result in hypoxia, which in subsequently contribute to VM formation. So, novel drugs have been under discovery to inhibit VM formation and phenotype transformation. For example, LY294002, a specific inhibitor of PI3K, is capable to inhibit the undifferentiation of embryonic melanoma cells to engage in VM on three‐dimensional typeI collagen matrices 86. Rapamycin, a HIF‐1a inhibitor, is able to decrease the expression of VEGF, VE‐cadherin and MMP‐2 to suppress VM formation. All these drugs targeting VM are capable to destroy the niche that maintains CSCs, block the metastasis passage of tumour cells, and reduce the recurrence of cancer.

## Conclusion

Epithelial‐mesenchymal transition is a dynamic biological process that converts epithelial cancer cells into dedifferentiated cells with additional mesenchymal properties. The hallmarks of EMT include loss of epithelial traits and the gain of mesenchymal phenotypes 14. Epithelial‐mesenchymal transition is also accompanied by increased expression of transcription factors including Twist, Snail, ZEB1 and ZEB2, which can bind to E‐cadherin promoter and suppress its transcription activity 15. Vasculogenic mimicry forms an ECM‐rich network and exposes tumour cells directly to blood flow, allow them to enter the microcirculation environment and metastasis to other organs, thus plays a crucial role in tumour growth and metastasis. Epithelial‐mesenchymal transition regulators including Twist, Snail and EMT‐related transcription factors are highly up‐regulated in VM‐forming tumour cells 5. These findings demonstrated that EMT plays a significant role in VM formation. Notably, compared with malignant tumour patients without VM, patients with VM have a worse prognosis 10. Vasculogenic mimicry is also one of the obstacles in the poor treatment of anti‐angiogenic drug for malignant tumour endothelial cells. Currently, more VM‐targeted therapies are found to inhibit EMT and VM formation for anticancer treatment and more drugs should be discovered for promising antitumour strategy in experimental and clinical research.

## Conflict of interest

The authors declare that we have no conflict of interest.
